# Bardoxolone and bardoxolone methyl, two Nrf2 activators in clinical trials, inhibit SARS-CoV-2 replication and its 3C-like protease

**DOI:** 10.1038/s41392-021-00628-x

**Published:** 2021-05-29

**Authors:** Qi Sun, Fei Ye, Hao Liang, Hongbo Liu, Chunmei Li, Roujian Lu, Baoying Huang, Li Zhao, Wenjie Tan, Luhua Lai

**Affiliations:** 1 BNLMS, Peking-Tsinghua Center for Life Sciences at College of Chemistry and Molecular Engineering, Peking University, Beijing, China; 2 NHC Key Laboratory of Biosafety, National Institute for Viral Disease Control and Prevention, Chinese Center for Disease Control and Prevention, China CDC, Beijing, China; 3 Center for Quantitative Biology, Academy for Advanced Interdisciplinary Studies, Peking University, Beijing, China

**Keywords:** Drug discovery, Chemical biology

**Dear Editor**,

The outbreak of coronavirus disease 2019 (COVID-19) has become a severe threat to global public health. Although many drug repurposing researches have been carried out, no effective drugs have been found in clinical studies.^1^ Among the viral proteins of the severe acute respiratory syndrome coronavirus 2 (SARS-CoV-2) that causes COVID-19, the 3C-like protease (3CL^pro^), the main protease responsible for viral polyprotein processing, is highly conserved among coronaviruses and serves as a promising target for broad-spectrum anti-CoV therapy. A number of SARS-CoV 3CL^pro^ inhibitors have been reported before. Recently, several SARS-CoV-2 3CL^pro^ inhibitors were discovered by structural-based drug design and high-throughput screening.^2^ Though these compounds showed encouraging antiviral activity in vitro, their in vivo efficacy, safety and metabolism need further investigation. There is a continuous and urgent need to discover new inhibitors with diverse chemical structures and novel mode of action. Many currently known coronavirus 3CL^pro^ inhibitors act through covalent binding.^2^ Covalent inhibitors are especially advantageous with enhanced therapeutic efficacy and minimized side effects, as exemplified by several approved anti-tumor covalent drugs.^3^ Thus, the screening of covalent 3CL^pro^ inhibitors, especially those with primary in vivo safety evaluation, may facilitate the discovery of antiviral agents.

We experimentally screened 315 compounds with electrophilic moieties that may covalently bind to the active site cysteine of SARS-CoV-2 3CL^pro^. Among them, 182 have been approved for clinical use and 51 have been processed to clinical trials. We found 15 compounds that inhibit SARS-CoV-2 3CL^pro^ activity with IC_50_’s of <30 μM (Fig. 1a, Supplementary Fig. S1a and Supplementary Table S1). We focused on the two compounds, bardoxolone methyl and bardoxolone, that have been processed to clinical trials and their anti-SARS-CoV-2 activity has not been reported before. The safety and pharmacokinetics of these two compounds are well characterized. We further tested the anti-SARS-CoV-2 activity of bardoxolone and bardoxolone methyl. With full-time incubation, bardoxolone methyl and bardoxolone inhibit SARS-CoV-2 replication in Vero cells with EC_50_ values of 0.29 μM (SI = 23.9) and 0.43 μM (SI = 56.6), respectively (Fig. 1b). Both compounds also inhibit the SARS-CoV-2 viral replication in human Calu-3 cells with EC_50_ values of 0.20 μM (SI = 5.8) and 0.42 μM (SI = 28.2), respectively. Efficacies were also confirmed with visualization of virus nucleoprotein (NP) expression using immunofluorescence microscopy at 48 h post infection (Fig. 1c).

**Fig. 1:**
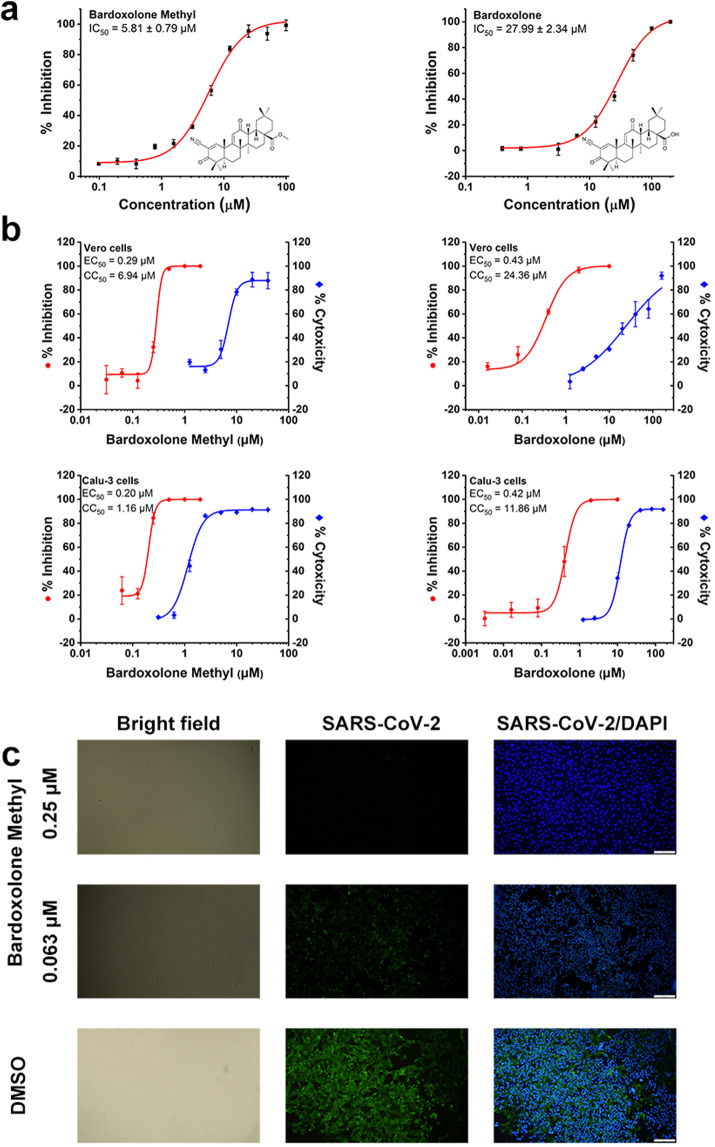
Inhibition activity of bardoxolone methyl and bardoxolone. **a** SARS-CoV-2 3CL^pro^ inhibition activity of bardoxolone methyl and bardoxolone. Various concentrations of bardoxolone methyl and bardoxolone were pre-incubated with SARS-CoV-2 3CL^pro^ for 30 min at room temperature before the addition of pNA-substrate. **b** Anti-SARS-CoV-2 activity and cytotoxicity of bardoxolone methyl and bardoxolone in Vero cells or Calu-3 cells. Cells were infected with SARS-CoV-2 at MOI of 0.01 (Vero cell line) and 1 (Calu-3 cell line) in the treatment of different doses of bardoxolone methyl and bardoxolone for 48 h. The viral yield in the cell supernatant was then quantified by qRT-PCR. The cytotoxicity of the compounds at different concentrations was measured by CCK-8 assays. The EC_50_ and CC_50_ were calculated by nonlinear regression analysis using Origin 2018 software. The selective indexes (SI) were calculated as the ratio of CC_50_ to EC_50_. **c** Immunofluorescence microscopy of virus infection upon treatment of bardoxolone methyl. Virus infection and drug treatment were performed as mentioned above. At 48 h post infection, the infected Vero cells were fixed, and then probed with mouse sera against the SARS-CoV-2 nucleoprotein as the primary antibody and Alexa 488-labeled goat anti-mouse IgG as the secondary antibody, respectively. The nuclei were stained with DAPI dye. Bars, 500 μm

To elucidate the mode of action of hit compounds against SARS-CoV-2 3CL^pro^, we performed enzyme kinetic studies with different concentrations of bardoxolone and bardoxolone methyl. In the absence of inhibitors, substrate cleavage increased with time. In contrast, in the presence of inhibitors, enzyme activity rapidly approached a plateau that is typical of covalent inhibition (Supplementary Fig. S2). We calculated the equilibrium dissociation constant (*K*i) and the inactivation rate constant (*k*_inact_) for these inhibitors. The *k*_inact_ values of bardoxolone and bardoxolone methyl are 0.00792 ± 0.00419 and 0.00218 ± 0.00018 s^−^^1^, respectively. Moreover, prolonged incubation of SARS-CoV-2 3CL^pro^ with the two compounds exhibited a time-dependent increase of inhibition activity (Supplementary Table S2 and Supplementary Fig. S3). Further liquid chromatography-tandem mass spectrometry (LC–MS/MS) analysis confirmed that bardoxolone binds SARS-CoV-2 3CL^pro^ in a reversible covalent manner (Supplementary Fig. S4). Molecular docking indicated that both bardoxolone methyl and bardoxolone bind to a pocket between domain I and domain II and form hydrogen bonds with Arg40 as well as hydrophobic interaction with Phe181 and Val186 (Supplementary Fig. S5). The sulfur atom of Cys85 is close to the reactive carbon atoms of bardoxolone methyl and bardoxolone with ~5 Å distance, suggesting the potential for covalent bond formation. The methyl group of bardoxolone methyl neutralizes the negative charge of bardoxolone, thus reduces its electrostatic repulsion with Glu55 and enhances its binding affinity.

Selective covalent inhibitors should specifically bind the targets in addition to covalent bond formation. We further used isothermal titration calorimetry to measure the thermodynamic binding parameters of bardoxolone and bardoxolone methyl with SARS-CoV-2 3CL^pro^ (Supplementary Fig. S6). All two compounds showed specific binding to SARS-CoV-2 3CL^pro^ with dissociation constants of 25.90 μM and 2.72 μM, respectively, which are in good agreement with their enzyme inhibition activity.

Bardoxolone and bardoxolone methyl are oleanolic acid-derived semi-synthetic triterpenoids that activate the Nrf2 pathway and inhibit the NF-κB pathway. Clinical trials are ongoing to explore the potential of bardoxolone methyl to treat chronic kidney diseases. The steady-state plasma concentration of bardoxolone by intravenous infusion exceeds 1 μM at doses below the maximum tolerated doses,^4^ which is well above EC_50_ value (0.43 μM) against SARS-CoV-2. It was reported that the Nrf2 pathway was suppressed in lung biopsies from COVID-19 patients and the induction of Nrf2 by 4-octyl-itaconate and dimethyl fumarate limited the host inflammatory response and inhibited the replication of SARS-CoV-2.^5^ Thus, the Nrf2 activators bardoxolone and bardoxolone methyl can be developed as a multifaceted antiviral treatment strategy by inhibiting viral replication, promoting resolution of inflammation, providing robust cyto-protection, and facilitating tissue repair. We recommend testing their activities as anti-COVID-19 agents.

## Supplementary information

Revised Supplementary Materials

## Data Availability

The data used and analyzed in this study are available in the main text and the Supplementary Materials.
