# Supplementary material for: Bardoxolone and bardoxolone methyl, two Nrf2 activators in clinical trials, inhibit SARS-CoV-2 replication and its 3C-like protease
Source: Signal Transduct Target Ther. 2021 May 29;6:212. doi: 10.1038/s41392-021-00628-x (PMC8164054; doi:10.1038/s41392-021-00628-x)
Supplement: Supplementary file 1 — Revised Supplementary Materials [file 41392_2021_628_MOESM1_ESM.pdf]

Supplementary Materials for

Bardoxolone and bardoxolone methyl, two Nrf2 activators in clinical trials, inhibit SARS-CoV-2 replication and its 3C-like protease

Qi Sun[1#], Fei Ye[2#], Hao Liang[1#], Hongbo Liu[1#], Chunmei Li[3], Roujian Lu[2], Baoying Huang[2], Li Zhao[2], Wenjie Tan[2*], Luhua Lai[1, 3*]

Correspondence to: lhlai@pku.edu.cn; tanwj28@163.com

This PDF file includes:

Materials and Methods
Supplementary Text
Figures. S1 to S6
Tables S1 to S3

Materials and Methods

Covalent compounds were purchased from Targetmol and Selleck Chemicals. HCV and HIV protease inhibitors were purchased from MedChemExpress and Targetmol. The Thr-Ser-Ala-Val-Leu-Gln-pNA and Dabcyl-KTSAVLQSGFRKM-Edans substrates were synthesized by GL Biochem. Vero cell and Calu-3 cell were purchased from ATCC.

Protein expression and purification

The recombinant SARS-CoV-2 $3CL^{pro}$ was expressed and purified as previously described[1].

Primary screening against SARS-CoV-2 $3CL^{pro}$

The inhibition assay of SARS-CoV-2 $3CL^{pro}$ activity was carried out as previously described[1]. Briefly, the activity of SARS-CoV-2 $3CL^{pro}$ was measured by a kinetics mode, with the substrate Thr-Ser-Ala-Val-Leu-Gln-pNA, using absorbance at 390 nm. The reaction buffer composed of 40 mM PBS, 100 mM NaCl, 1 mM EDTA, 0.1% Triton X-100, pH 7.3 was used. For the preliminary screening and IC_{50} measurements, 0.5 μM protease was incubated with inhibitor at room temperature for 30 min, and then the reaction was initiated by adding 200 μM substrate, the reaction was recorded for 20 min. Among all the 315 compounds that we tested, 15 showed inhibition activity over 50% at 50 μM. IC_{50} values were further analyzed and fitted with Hill 1 function of Origin 2018.

Proteolytic kinetic studies and time-dependent inhibitory measurement

Proteolytic reaction progress curve kinetics measurements with bardoxolone, bardoxolone methyl, and Z-DEVD-FMK were carried out as follows: 0.5 μM protease was added to 20 μM fluorescent substrate (Dabcyl-KTSAVLQSGFRKM-Edans) with various concentrations of testing inhibitors in reaction buffer to initiate the reaction. The fluorescence signal generated by the cleavage of the substrate was monitored for 2 h at an emission wavelength of 460 nm with excitation at 360 nm, using a plate reader (Synergy, Biotek). The equilibrium dissociation constant for inhibitor (Ki) and inactivation rate constant (k_{inact}) are calculated according to published report[2].

For time-dependent inhibitory measurement, various concentrations of inhibitors were pre-incubated with SARS-CoV-2 $3CL^{pro}$ for different time at room temperature before the addition of fluorescent substrate. The fluorescence signal generated by the cleavage of the substrate was recorded for 20 min. The percent of inhibition was calculated by Vi/V0, where V0 and Vi represent the mean reaction rate of the enzyme incubated with DMSO or inhibitors. IC_{50} was fitted with Hill1 function of Origin 2018.

Mass spectrometry analysis

10 μL compounds (4 mM in DMSO) were added into 200 μL SARS-CoV-2 $3CL^{pro}$ (100 μM) in enzyme activity assay buffer. The mixtures were incubated in room temperature for 2 h. Then exchanged the buffer into deionized water by ultrafiltration. The samples were assayed in positive-ion mode with a QTRAP mass spectrometer (AB Sciex 5500, USA) for detecting the molecular weight of intact proteins. For tandem mass spectrometry analysis, the samples were precipitated and redissolved by 8 M urea, and then digested by chymotrypsin and analyzed by LC-MS/MS (Orbitrap Elite, USA).

Molecular docking

The crystal structure of SARS-CoV-2 $3CL^{pro}$ in complex with an inhibitor N3 was used (PDB ID: 6LU7). A grid box large enough to encompass the entire 3CL protein was created. Polar hydrogens and Gasteiger partial charges were added to proteins and ligands by AutoDockTools[3] Molecular docking was performed using Vina[4].

Isothermal titration calorimetry determination

Isothermal titration calorimetry experiments were carried out using an iTC200 calorimeter (General Electric Co.). SARS-CoV-2 $3CL^{pro}$ and the inhibitors were dissolved in buffer composed of 40 mM PBS, 100 mM NaCl, pH 7.3, and 1 mM EDTA with 2% DMSO. The titration was performed at 25℃ by injecting aliquots of protease solution (0.8-1.5 mM) into the calorimetric cell containing the inhibitor at a concentration of 40-75 μM, with stirring at 1000 rpm. All experiments were executed using an initial injection of 0.4 μL followed by 19 injections of 2 μL with 4s duration each and a 240s interval between injections. The heat associated with inhibitor binding was obtained by subtracting the heat of dilution from the heat of reaction.

Cytotoxicity and antiviral assays

For cytotoxicity assays, about 2×10^4 Vero cells or 1×10^4 Calu-3 cells were seeded into 96-well plates. The next day, various concentration of drugs was added to each well. After 48 h, the relative numbers of surviving cells were measured by CCK8 (DOJINDO, Japan). For antiviral assays in Vero cells, a clinical isolated SARS-COV-2 virus (C-Tan-nCoV Wuhan strain 01) was propagated in Vero cells. Pre-seeded Vero cells (2×10^4 cells/well) were pre-treated with indicated concentrations of test drugs for 1 h, and the virus (MOI of 0.01) was subsequently added to allow infection for 1 h at 37℃. For antiviral assays in Calu-3 cells, cells were pre-treated with indicated concentrations of test drugs for 1 h, then adsorbed with a MOI of 1 PFU per cell of SARS-CoV-2 (C-Tan-nCoV Wuhan strain 01) for 1 h. Then, the virus-drug mixture was removed and the cells were washed with DMEM and further treated with medium contained drugs at various concentrations for 48h. The cell supernatant was collected and the RNA was extracted and analyzed by relative quantification using RT-PCR as in the previous study[1].

Immunofluorescence microscopy

Pre-seeded Vero cells (2×10^4 cells/well) were pre-treated with indicated concentrations of test drugs for 1 h, and the virus (MOI of 0.01) was subsequently added to allow infection for 1 h at 37℃. At 48 h post infection, the infected cells were fixed, and then probed with mouse sera against the SARS-COV-2 N protein (ABMAX, cat#05-0150) as the primary antibody and Alexa 488-labeled goat anti-mouse IgG (1:1000, Invitrogen) as the secondary antibody, respectively. The nuclei were stained with DAPI dye. Bars, 500 μm.

Supplementary Text

The SARS-CoV-2 $3CL^{pro}$ inhibition activity of HCV and HIV protease inhibitors

Boceprevir and telaprevir are peptide mimic inhibitors that covalently inhibit the activity of HCV NS3/NS4 protease. It was suggested that HIV-1 and HCV protease inhibitors might be useful as anti-SARS-CoV-2 agents. We further tested another 15 approved HIV-1 protease and HCV NS3/NS4A inhibitors against SARS-CoV-2 $3CL^{pro}$ (Table S3). In fact, among these peptide mimics viral protease inhibitors, only covalent inhibitors (boceprevir, telaprevir and narlaprevir) can inhibit SARS-CoV-2 $3CL^{pro}$ activity, while all the non-covalent inhibitors cannot, supporting the rationality of screening covalent inhibitors for SARS-CoV-2 $3CL^{pro}$.

LC-MS/MS analysis of hit compounds with SARS-CoV-2 $3CL^{pro}$

We also carried out liquid chromatography-tandem mass spectrometry (LC-MS/MS) analysis to verify whether bardoxolone and Z-DEVD-FMK bind SARS-CoV-2 $3CL^{pro}$ in covalent manner. Intact protein mass spectrometry study confirmed that compound Z-DEVD-FMK completely modified SARS-CoV-2 $3CL^{pro}$, detected as a main peak with a MW shift of +649 Da ($C_{30}H_{40}N_4O_{12}$), which is equal to the mass of the SARS-CoV-2 $3CL^{pro}$ / Z-DEVD-FMK complex along with the removal of hydrogen fluoride (Figure S4). Furthermore, tandem MS/MS analysis demonstrated that Z-DEVD-FMK covalently bound to the catalytic site C145 of SARS-CoV-2 $3CL^{pro}$. A trebly charged fragment at m/z 1076.159 was identified from the sample treated with Z-DEVD-FMK, corresponding to the modified peptide QC (carbamidomethyl)AMRPNFTIKGSF LNGSC($C_{30}H_{40}N_4O_{12}$)GSVGF. However, bardoxolone could only partially modify SARS-CoV-2 $3CL^{pro}$ and the modified peptide was not detected, perhaps due to the instability of reversible covalent bond under enzymatic cleavage condition[5].

Predicted binding mode of bardoxolone methyl and bardoxolone

We performed blind docking to thoroughly explore the possible binding mode. A grid box large enough to encompass the entire 3CL protein was created to thoroughly explore the binding site. As shown in Figure S5a and S5b, bardoxolone methyl and bardoxolone bind to SARS-CoV-2 $3CL^{pro}$ in a similar way. Both of them form hydrogen bonds with Arg40 and hydrophobic interaction with Phe181 and Val186. Both bardoxolone and bardoxolone methyl contain two α, β unsaturated ketones in the A and C ring, respectively, that may serve as the site of Michael addition reaction. The distances between the sulfur atom of Cys85 and the reactive carbon atom of bardoxolone methyl and bardoxolone are ~ 5 Å, suggesting the potential for covalent bond formation. Electrostatic surface analysis indicated that the methyl group of bardoxolone methyl neutralizes the negative charge of bardoxolone, thus reduces its electrostatic repulsion and enhances its binding affinity.

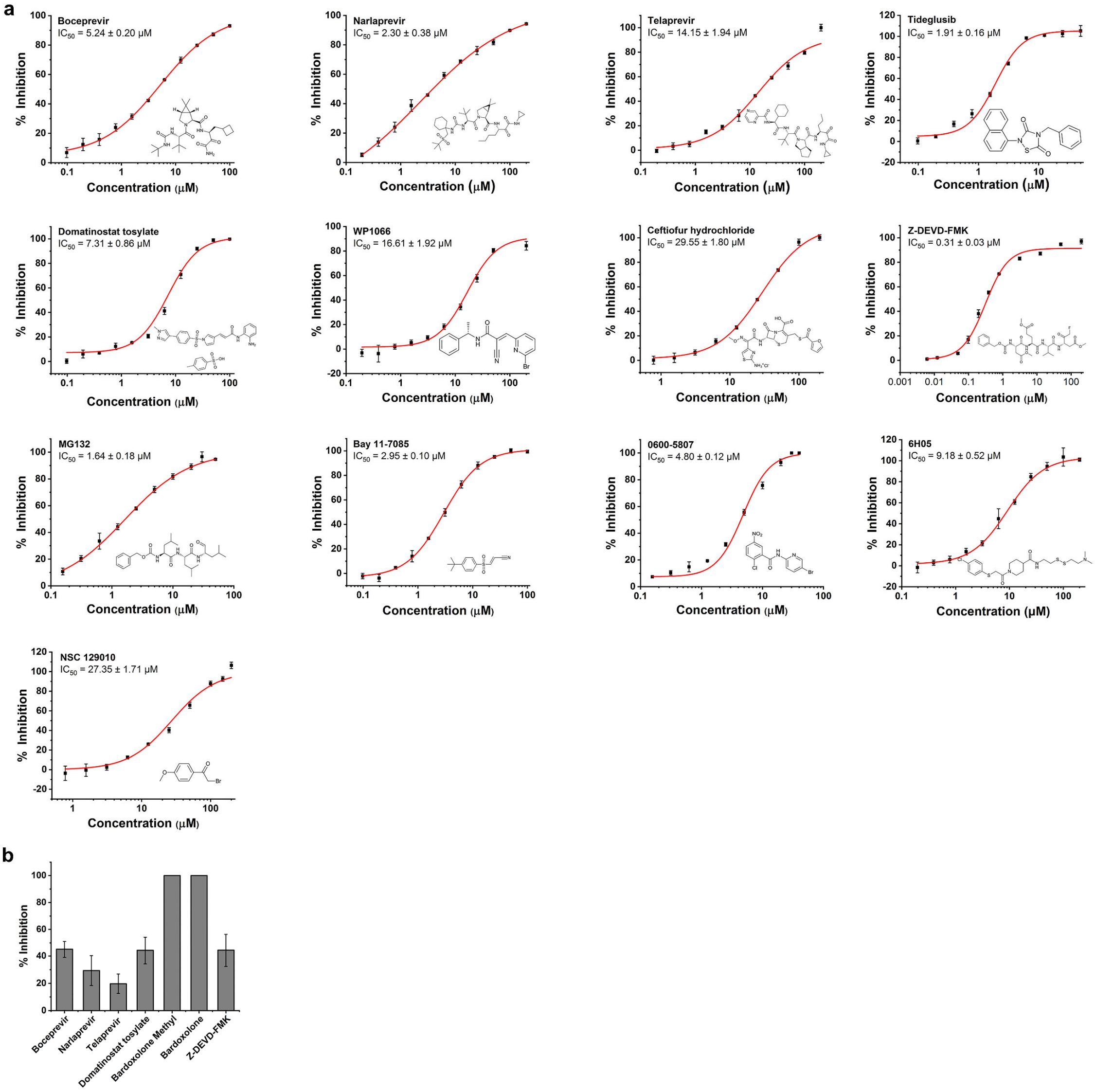

Figure. S1.

a SARS-CoV-2 3CL^{pro} inhibition activity of active compounds. Various concentrations of hits were pre-incubated with SARS-CoV-2 3CL^{pro} for 30 min at room temperature before the addition of pNA-substrate. Value = means ± SE from three independent experiments. b The anti–SARS-CoV-2 activity of test compounds at 10 μM or 1 μM (bardoxolone methyl and bardoxolone) treatment in Vero cells was measured by qRT-PCR assay. Value = means ± SE from three independent experiments.

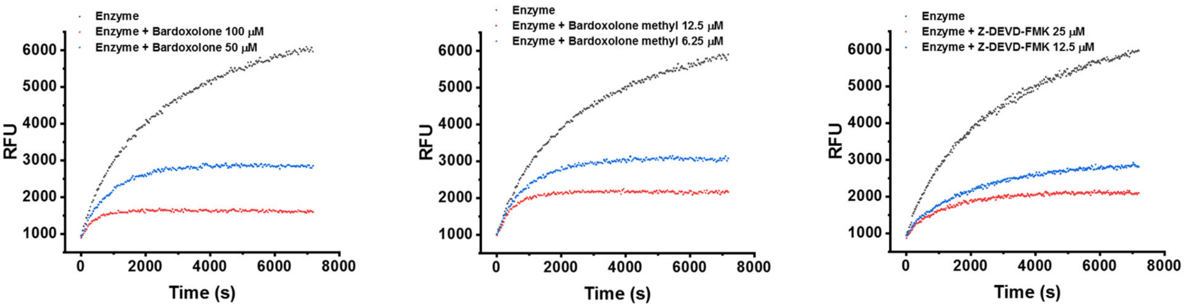

Figure. S2.

Kinetic reaction progression curves of SARS-CoV-2 3CL^{pro} in the absence or the presence of compounds. In the kinetic studies, 20 μM FRET substrate was added to a solution containing 0.5 μM protease and various concentrations of inhibitors to initiate the reaction, the reaction was then monitored for 2 hrs.

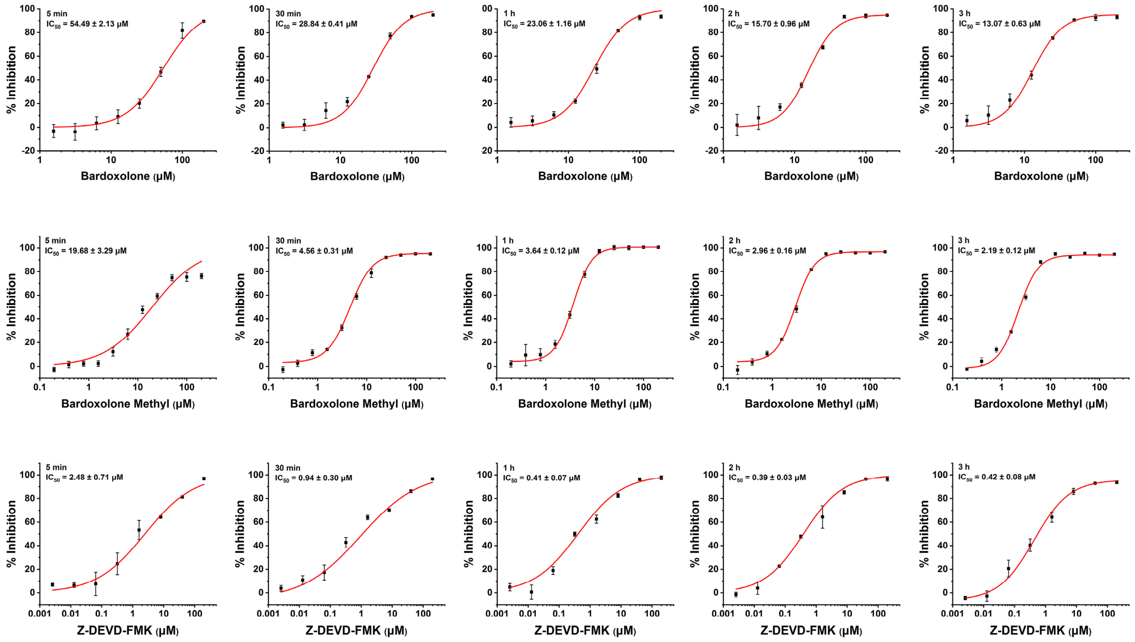

Figure. S3.

The dose–response curves of bardoxolone (a), bardoxolone methyl (b) and Z-DEVD-FMK (c) against SARS-CoV-2 $3CL^{pro}$ at various incubation times. Value = means ± SE from three independent experiments.

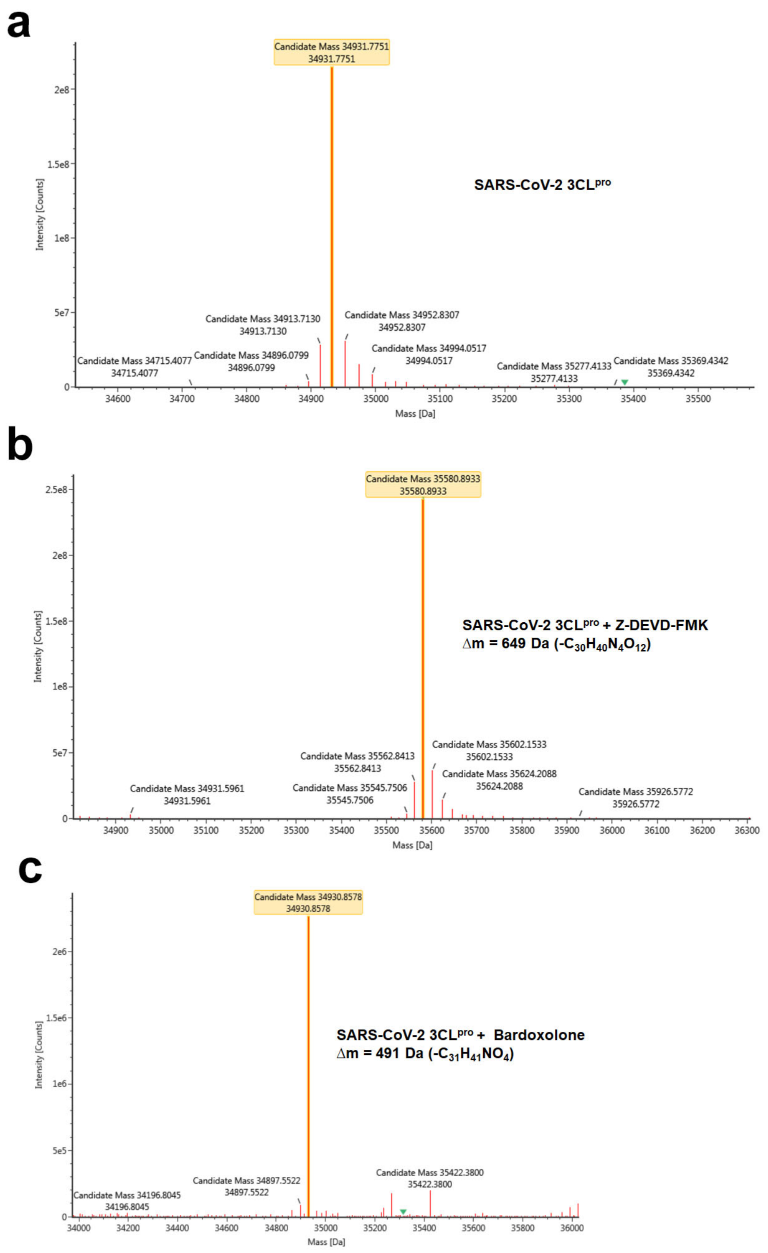

Figure. S4.

LC-MS/MS analysis of SARS-CoV-2 3CL^{pro} in the absence (a) or the presence of compound Z-DEVD-FMK (b) and bardoxolone (c).

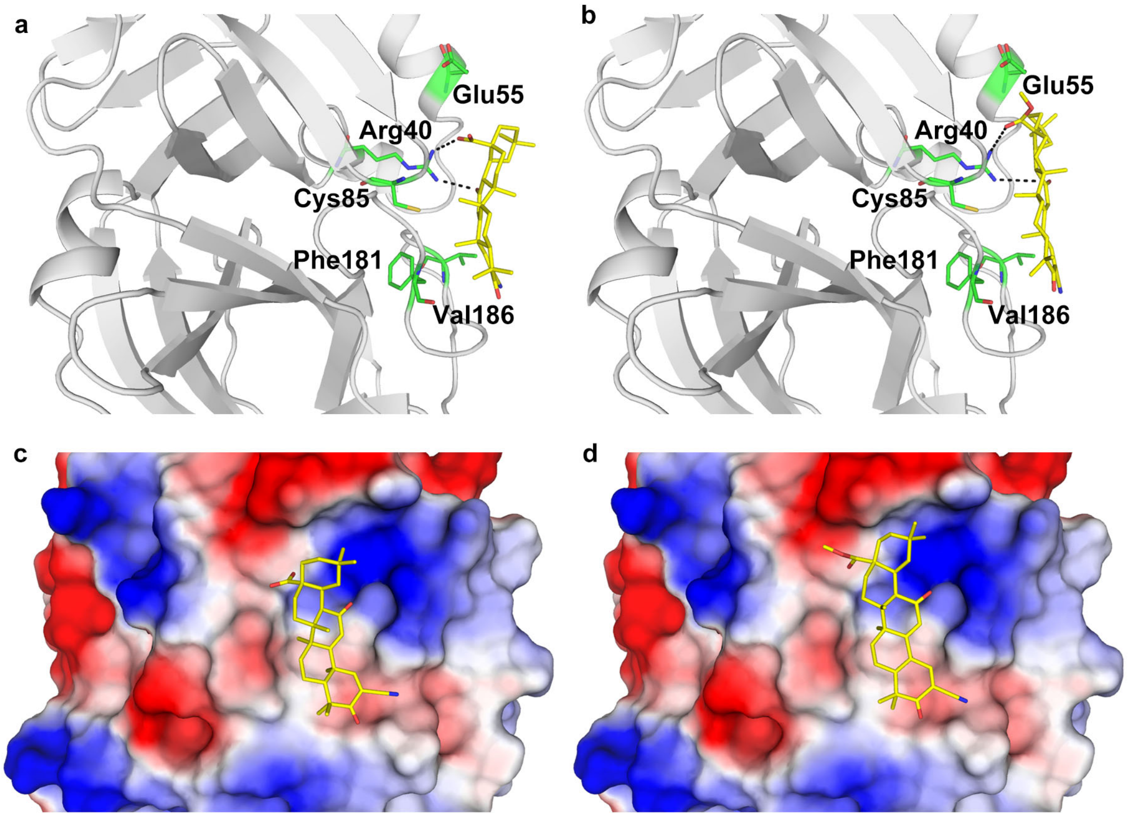

Figure. S5.

The docking model of SARS-CoV-2 3CL^{pro} and bardoxolone methyl (a, c), bardoxolone (b, d). Ligands are shown as yellow sticks, and the SARS-CoV-2 3CL^{pro} is displayed as cartoons and electrostatic surface. Both compounds form hydrogen bonds with Arg40 and hydrophobic interaction with Phe181 and Val186. The two α, β unsaturated ketones in the A and C ring in the compounds may serve as the site of Michael addition reaction. The distances between the sulfur atom of Cys85 and the reactive carbon atom of bardoxolone methyl and bardoxolone are about 5 Å, suggesting potential covalent bond formation. The methyl group of bardoxolone methyl neutralizes the negative charge of bardoxolone, thus reduces its electrostatic repulsion and enhances its binding affinity (c, d).

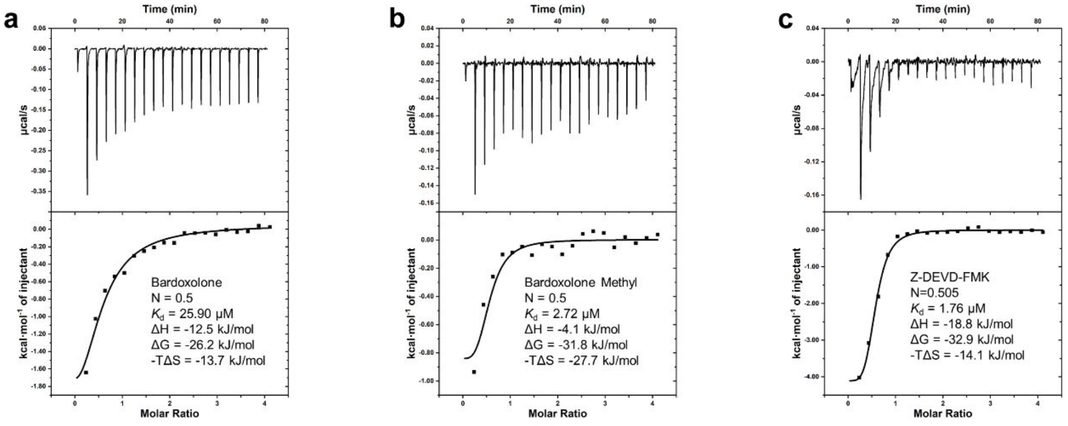

Figure. S6.

The binding ability of bardoxolone (a), bardoxolone methyl (b) and Z-DEVD-FMK (c) with SARS-CoV-2 $3CL^{pro}$ characterized by ITC.

Table S1.
SARS-CoV-2 3CL^{pro} inhibition activity of active compounds.

Compound	SARS-CoV-2 3CL^{pro} IC$_{50}$ (μM)*	Development stage
Boceprevir	5.24 ± 0.20	FDA-approved
Narlaprevir	2.30 ± 0.38	In clinical trials
Telaprevir	14.15 ± 1.94	FDA-approved
Bardoxolone methyl	5.81 ± 0.79	In clinical trials
Bardoxolone	27.99 ± 2.34	In clinical trials
Tideglusib	1.91 ± 0.16	In clinical trials
Domatinostat tosylate	7.31 ± 0.86	In clinical trials
WP1066	16.61 ± 1.92	In clinical trials
Ceftiofur hydrochloride	29.55 ± 1.80	Veterinary
Z-DEVD-FMK	0.31 ± 0.03	Preclinical
MG132	1.64 ± 0.18	Preclinical
Bay 11-7085	2.95 ± 0.10	Preclinical
0600-5807	4.80 ± 0.12	Preclinical
6H05	9.18 ± 0.52	Preclinical
NSC129010	27.35 ± 1.71	Preclinical

* Various concentrations of hits were pre-incubated with SARS-CoV-2 3CL^{pro} for 30 min at room temperature before the addition of pNA-substrate. Value = means ± SE from three independent experiments.

Table S2.

Time-dependent inhibition of bardoxolone, bardoxolone methyl and Z-DEVD-FMK against SARS-CoV-2 3CL^{pro}.

Incubation time	Bardoxolone IC_{50} (µM)*	Bardoxolone methy IC_{50} (µM) *	Z-DEVD-FMK IC_{50} (µM) *
5 min	54.49 ± 2.13	19.68 ± 3.29	2.48 ± 0.71
30 min	28.84 ± 0.41	4.56 ± 0.31	0.94 ± 0.30
1 h	23.06 ± 1.16	3.64 ± 0.12	0.41 ± 0.07
2 h	15.70 ± 0.96	2.96 ± 0.16	0.39 ± 0.03
3 h	13.07 ± 0.63	2.19 ± 0.12	0.42 ± 0.08

* Various concentrations of inhibitors were pre-incubated with SARS-CoV-2 3CL^{pro} for different time at room temperature before the addition of fluorescent substrate. Value = means ± SE from three independent experiments.

Table S3.
The SARS-CoV-2 $3CL^{pro}$ inhibition activity of HCV and HIV protease inhibitors.

Drug	Indication	Inhibition (%, 50 μM)
Atazanavir sulfate	HIV infection	3.49 ± 6.15
Ritonavir	HIV infection	1.61 ± 0.12
Lopinavir	HIV infection	14.20 ± 5.67
Darunavir	HIV infection	4.79 ± 0.65
Nelfinavir Mesylate	HIV infection	35.93 ± 4.91
Amprenavir	HIV infection	1.09 ± 0.82
Indinavir sulfate	HIV infection	1.71 ± 0.13
Saquinavir	HIV infection	1.36 ± 0.22
Tipranavir	HIV infection	11.18 ± 1.23
Paritaprevir	HCV infection	1.31 ± 1.79
Danoprevir	HCV infection	0.05 ± 0.99
Asunaprevir	HCV infection	4.26 ± 0.87
Simeprevir	HCV infection	1.69 ± 1.86
Glecaprevir	HCV infection	4.09 ± 1.65
Grazoprevir	HCV infection	4.86 ± 0.33
Velpatasvir	HCV infection	2.16 ± 0.64*
Ledipasvir	HCV infection	1.62 ± 0.39*

* Due to the low water solubility, velpatasvir and ledipasvir were measured at 12.5 μM.

References

1 Liu, H. *et al.* Scutellaria baicalensis extract and baicalein inhibit replication of SARS-CoV-2 and its 3C-like protease in vitro. *J Enzyme Inhib Med Chem*. **36**, 497-503 (2020).

2 Yang, H. *et al.* Design of wide-spectrum inhibitors targeting coronavirus main proteases. *PLoS Biol.* **3**, e324 (2005).

3 Morris, G. M. *et al.* AutoDock4 and AutoDockTools4: Automated docking with selective receptor flexibility. *J Comput Chem.* **30**, 2785-2791 (2009).

4 Trott, O. & Olson, A. J. AutoDock Vina: improving the speed and accuracy of docking with a new scoring function, efficient optimization, and multithreading. *J Comput Chem*. **31**, 455-461 (2010).

5 Meng, X. *et al.* CDDO-imidazolide Targets Multiple Amino Acid Residues on the Nrf2 Adaptor, Keap1. *J Med Chem*. **63**, 9965-9976 (2020).
